# Protective effect of exclusive breastfeeding against hand, foot and mouth disease

**DOI:** 10.1186/s12879-014-0645-6

**Published:** 2014-12-04

**Authors:** Hualiang Lin, Limei Sun, Jinyan Lin, Jianfeng He, Aiping Deng, Min Kang, Hanri Zeng, Wenjun Ma, Yonghui Zhang

**Affiliations:** Guangdong Provincial Institute of Public Health, Guangdong Provincial Center for Disease Control and Prevention, Guangzhou, 511430 China; Guangdong Provincial Center for Disease Control and Prevention, 160, Qunxian Road, Panyu Guangzhou, 511430 China

**Keywords:** Exclusive breastfeeding, Hand, foot and mouth disease, Children, China

## Abstract

**Background:**

Infants who are exclusively breastfed receive natural protection against some infectious agents. This study examined whether there was protective effect of exclusive breastfeeding on the occurrence of hand, foot and mouth disease, which was an emerging infectious disease among children in China.

**Methods:**

A community-based case–control study was carried out among children age 4 years or younger in Guangdong Province, China. Cases were newly diagnosed hand, foot and mouth disease. Controls were randomly sampled from healthy children from the nearby village. Unconditional logistic regression model was used to estimate the odds ratio (OR) for exclusive breastfeeding after adjusting for potential confounding factors.

**Results:**

A total of 316 cases and 566 controls were included in the analysis. Significantly beneficial effect of exclusive breastfeeding during the first 6 months was observed for hand, foot and mouth disease occurrence. The overall OR was 0.63 (95% CI: 0.47-0.85) for exclusive breastfeeding compared with mixed feeding type. The age-specific analyses indicated that the protective effect persisted till the age of 28 months.

**Conclusions:**

This study suggests that exclusive breastfeeding might have protective effect against HFMD infection among the children within 28 months of age.

**Electronic supplementary material:**

The online version of this article (doi:10.1186/s12879-014-0645-6) contains supplementary material, which is available to authorized users.

## Background

In 2001, the World Health Organization (WHO) recommended that infants should be exclusively breastfed for six months [1]. However, not every member state complied with this recommendation. Substantial evidence has indicated that early nutrition is beneficial for long term health, by programming aspects of subsequent cognitive function, obesity, risk of cardiovascular disease, cancer, and atopy [2]. However, the evidence is still lacking on the protective effect of exclusive breastfeeding at early life time, especially on infectious diseases, and how long the protective effect can persist [3].

Hand, foot and mouth disease (HFMD) is a common infectious disease among children, mainly caused by the Enterovirus 71 and Coxsackievirus A 16 [4]. In most times this infection is mild and self-limiting, however more severe clinical symptoms may occur when there are complications, such as encephalitis, aseptic meningitis, and acute flaccid paralysis [5],[6]. Asia-Pacific countries have experienced an increasing trend of HFMD outbreaks in the past decades, resulting in thousands of deaths among the children [7]. For example, a total of 1,619,706 new HFMD cases with 509 deaths were reported in China in the year of 2011 [8]. Severe outbreaks have also been witnessed in Taiwan in 1998 with 129,106 HFMD cases and 78 children deaths being reported [9].

There is no specific drug or effective vaccine available for HFMD, so preventive measures such as avoiding direct contact with infective persons, disinfection of viral contaminated environment, and good personal hygiene habits remain the only effective way to prevent its transmission [10],[11]. However, few studies have been conducted to examine the underlying risk factors of this disease [12],[13]. In Taiwan, a case–control study suggested that age, attendance at kindergartens/children care centers, contacts with HFMD cases, greater number of children in a family were risk factors of this illness among pre-school children [14]. A similar case–control study in Zhejiang Province, China found that playing with neighborhood children, visiting an outpatient clinic, and community exposures to crowded places were potential risk factors of HFMD [15].

Some studies have reported that exclusive breastfeeding can reduce the risk of many infectious diseases and other diseases in children, such as otitis media, gastroenteritis, necrotizing enterocolitis, respiratory diseases, sudden infant death syndrome, obesity, and hypertension [16],[17]. And in some countries, exclusive breastfeeding during the first 6 months has been reported to have a protective effect against gastrointestinal tract infections in infancy [18],[19]. One recent study examined the effect of exclusive breastfeeding on fever occurrence in HFMD patients, showing that children HFMD patients with exclusive breastfeeding had a lower risk of fever in Xi’an, China [12].

The present study used a community-based case–control study in Guangdong Province, China to examine the association between exclusive breastfeeding during the first 6 months and risk of hand, foot and mouth disease among the children under 4 years older, and we also examined how long the effect can persist by age-specific analyses.

## Methods

### Study design

**Figure 1:**
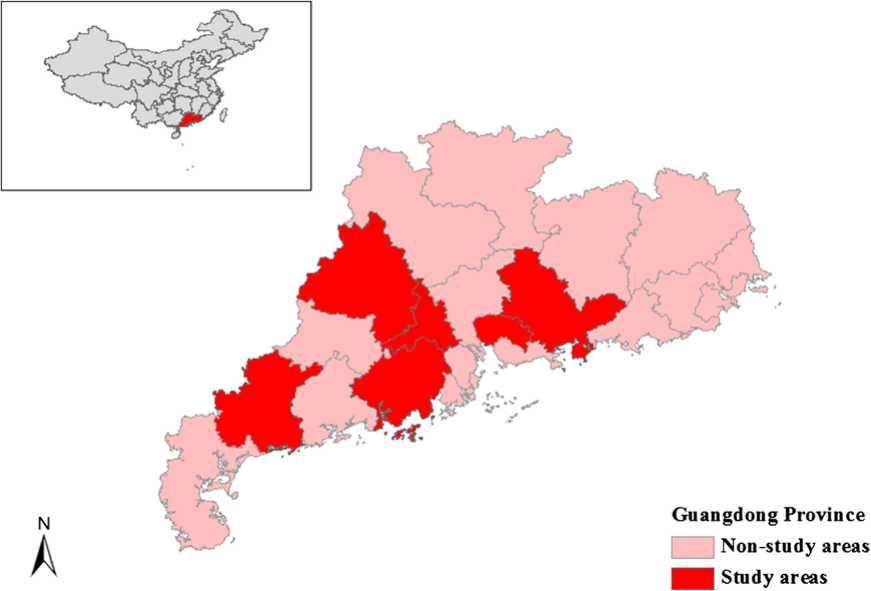
**Location of the study area in China.**

We used information collected from a case–control study conducted in 6 cities in Guangdong Province, China (Figure 1), regarding risk factors for children hand, foot and mouth disease. The survey was conducted during the period from June 2010 to December 2011. These cities account for approximately 19% of the population and 23% of the province’s geographic area. Approval to conduct this study was granted by the Medical Ethics Committee of Guangdong Provincial Centre for Disease Control and Prevention. Informed consent was obtained before each interview.

A list of new diagnosed cases of hand, foot and mouth disease were provided by local hospitals, eligible cases were those aged 4 years or younger, who were newly diagnosed by a clinical doctor within 7 days before the field interview. And this study was restricted to children who have not attended to a kindergarten or child care center, and resided in the study location for at least 6 months. It was estimated that about 30% of the new diagnosed cases were randomly selected from the patient list and included in this analysis. The diagnosis was based on the Hand, Foot and Mouth Disease Clinic Guide (2010 edition) issued by the Ministry of Health of China [20]. Hand, foot and mouth disease is characterized by a brief febrile illness in children and typical skin rash, with or without mouth ulcers. Typically, the rash is papulovesicular and affects the palms or soles of the feet, or both [21].

We got the list of the potentially eligible children under 4 years of age from a neighbor village and 2–3 controls were randomly selected when one case was determined, these villages should not have HFMD cases one year before this survey. The eligible controls should have not been diagnosed as a hand, foot and mouth disease, or any other gastrointestinal tract diseases 6 months before the survey and should have no direct contacts with any cases. In order to avoid any misclassification, the feces of the controls were collected and underwent enterovirus RT-PCR test, if any control was positive for EV71, CA16 and other enterovirus, he/she would be excluded from the analysis. Details about the laboratory testing can be found elsewhere [22].

Face-to-face interviews were conducted at the subjects’ home by trained interviewers using a structured questionnaire. Interviews were administered to the guardians of the subjects, usually their parents. In addition to detailed information about the breastfeeding type at the first 6 months of the children, information was also collected for demographic factors, household information, contacts with HFMD patients, and hand washing habits. Exclusive breastfeeding was defined as excluding solids or any other fluids (including infant formulas) except medicines, vitamins, and minerals, in this study we found that the feeding type was either exclusive breastfeeding or mixed type of breast feeding and supplementary fluids, so we used the mixed feeding type as the reference group in the analysis. We asked about the exposure during 2 weeks before onset of the symptom of HFMD for cases or before the survey for controls, as this infection usually has incubation period of about 1–2 weeks [23],[24]. For example, the subjects were asked whether they went to see a doctor for any illness or whether they went to visit a patient in a hospital or at the patient’s home. We also included the information of the children’s hand-washing habits before dinner, after toilet use and the guardians’ hand washing before contacting with the child. The information about the contacting with other children in the playgrounds indoors and outdoors were also included in this survey. We also included some household factors in the questionnaire, such as monthly household income, household hygiene condition, house area, and number of family members. The household hygiene condition was evaluated using an indicator of whether there was any visible trash around the house of the subjects. We calculated the crowding of the family by dividing the family numbers for per 100 m^2^ house area.

We recruited and trained public health doctors from community health centers to conduct the interviews. If one subject was not convenient for the interview, one revisit was attempted. As it was unlikely for the interviewers to be blinded to the case/control status, they were unaware of the main study hypothesis and were trained to administer strictly the questionnaires in an equal manner to the cases and controls.

### Statistical analysis

All data were double entered into a database using Epidata software. The χ^2^ tests or t tests were used to examine the differences of socio-demographic factors between the cases and controls. Odds ratios (ORs) and 95% confidence intervals (95% CIs) were estimated for the breastfeeding type using unconditional logistic regression models. Univariate analysis was performed for each potential confounding factor first. Variables that had a p value of less than 0.10 in the univariate analyses were included in the multivariate logistic regression model as adjustment variables [25]. The correlations between the potential confounding factors were also examined, if two variables were highly correlated with each other, they would be not included in the same model to avoid the effect of co-linearity [25].

To examine the duration of the protective effect of exclusive breastfeeding, we did a subgroup analysis for different age groups with 6 months as the interval (<6 months, 6–12 months, 13–18 months, 19–24 months, 25–30 months, 30–48 months), when one age group was found to be non-significant, a further analysis would be conducted for each month within that age group to find the cut-point value.

All the data management and statistical analyses were performed using the R software [26]. P value <0.05 was considered statistically significant.

## Results

During the study period, a total of 316 HFMD cases and 621 controls were originally enrolled in this study, among which, 55 controls were found to be enterovirus positive and thus excluded from the subsequent analyses.

The distribution of socio-demographic factors and other variables among the cases and controls was shown in Table 1. Cases were statistically older than controls (1.7 versus 1.6 years, P = 0.01 for t test), however the distribution of the age group was not statistically different among the two groups. Fewer cases than controls were fed with only breast milk (43.0% versus 55.8%, P = 0.004 for χ^2^ test). The cases and controls differed significantly in the following variables: hand washing habits, guardians’ hand washing before contacting with the child, going to indoor or outdoor playground, and household environment. There were no significant differences between the cases and controls in height, weight, house crowdedness, household income, sex, whether having gone to see a doctor, or whether having visited a patient before the onset of the symptom or the survey.

**Table 1:** **Comparison of major risk factors between cases and controls**

Factor	Control (n = 566) n (%)	Case (n = 316) n (%)	P value *
Age (yrs)	1.6	1.7	0.01
< 6 m	125(22.1)	49(15.5)	
6-12 m	93(16.4)	60(19.0)	
13-18 m	97(17.1)	50(15.8)	
19-24 m	75(13.3)	51(16.1)	
25-28 m&	41(7.2)	32(10.1)	
29-48 m	135(23.9)	74(23.4)	0.12
Height (cm)	75.9	79.5	0.10
Weight (kg)	11.0	11.3	0.25
Sex			
Male	329(58.1)	200(63.3)	
Female	237(41.9)	116(36.7)	0.15
Breastfeeding type			
Mixed	250(44.2)	180(57.0)	
Exclusive breastfeeding	316(55.8)	136(43.0)	0.004
Went to see a doctor			
No	518(94.5)	296(93.7)	
Yes	30(5.5)	20(6.3)	0.74
Went to visit a patient			
No	458(83.6)	255(80.7)	
Yes	90(16.4)	61(19.3)	0.33
Hand-washing before dinner			
Yes	435(78.8)	182(58.0)	
No	117(21.2)	132(42.0)	0.001
Hand-washing after toilet use			
Yes	375(70.0)	182(58.7)	
No	161(30.0)	128(41.3)	0.001
Hand-washing before contacting child			
No	207(38.6)	170(55.6)	
Yes	329(61.4)	136(44.4)	0.001
Went to indoor playground			
No	462(84.5)	226(71.5)	
Yes	85(15.5)	90(28.4)	0.001
Went to outdoor playground			
No	441(80.6)	206(65.2)	
Yes	106(19.4)	110(34.8)	0.001
House trash			
No	515(91.0)	239(75.6)	
Yes	51(9.0)	77(24.4)	0.001
Monthly household income (RMB)			
<10 k	281(84.4)	153(85.5)	
≥10 k	52(15.6)	26(14.5)	0.84
House crowding (person/100 m^2^)	6.3	6.9	0.14

*χ^2^ test for categorical variables and t test for continuous variables.

The crude and adjusted ORs and 95% CIs for children breastfeeding type were illustrated in Table 2. In the univariate analysis, we found that exclusive breastfeeding was associated with lower risk of HFMD (OR = 0.60, 95% CI: 0.45-0.79) with mixed breastfeeding as the reference. Our correlation analysis for the potential confounding factors suggested that going to indoor playground was significantly associated with going to outdoor playground, and hand-washing before dinner was significantly associated with hand-washing after toilet use, so in the multivariate models, we include only one variable (going to outdoor playground and hand-washing before dinner) of these two pairs of factors. In the sensitivity analysis, we included other variables, which yielded similar effect estimates.

**Table 2:** **Crude and adjusted ORs for hand, foot and mouth disease related to exclusive breastfeeding**

	OR (95%CI)			
	Crude	P value	Adjusted*	P value
Overall	0.60 (0.45-0.79)	0.001	0.63 (0.47-0.85)	0.002
Sex				
Males	0.61 (0.43-0.87)	0.01	0.65 (0.44-0.95)	0.03
Females	0.59 (0.38-0.93)	0.02	0.54 (0.33-0.89)	0.02
Age				
< 6 m	0.25 (0.01-1.88)	0.23	0.31 (0.01-3.39)	0.38
6-12 m	0.58 (0.26-1.06)	0.08	0.45 (0.22-0.90)	0.03
13-18 m	0.59 (0.28-1.03)	0.06	0.61 (0.24-0.99)	0.04
19-24 m	0.47 (0.25-0.87)	0.02	0.38 (0.18-0.77)	0.01
25-28 m&	0.41 (0.18-0.92)	0.03	0.41 (0.18-0.92)	0.03
29-48 m	1.10 (0.59-2.00)	0.78	1.19 (0.62-2.30)	0.60

*In multivariate logistic regression model, we controlled for age, hand washing habit before dinner, whether the children have played in outdoor playgrounds, household environment, etc. & Our initial analysis used 6 month interval for the age group-specific analysis, we found the group of 25–30 months was not statistically significant, in order to check whether month was the cut-off point, we further examined the association for each month within this age group, and found it was not significant after 28 months.

Compared with mixed feeding type, children with exclusive breastfeeding had significantly lower risk of HFMD (OR = 0.63, 95% CI: 0.47-0.85) after controlling for potential confounding factors. The association was similar for males and females. It seemed that the exclusive breastfeeding was not protective for all age groups, though the OR for those aged younger than 6 months was smaller than 1, but it was not statistically significant. The results showed that it had beneficial effect for those aged 6 months to 28 months. And adjusting for various confounding factors did not change the risk estimation substantially.

## Discussion

To our knowledge, our study might be the first study linking exclusive breastfeeding with the risk of hand, foot and mouth disease, an emerging infectious disease in China, particularly among children. The current study added to the knowledge gap in Chinese population that exclusive breastfeeding could prevent the occurrence of hand, foot and mouth disease, and this protection could persist for about 28 months.

Our finding of the protective effect of exclusive breastfeeding against HFMD was consistent with a few previous studies, which also found a beneficial effect of exclusive breastfeeding for infectious diseases [18],[27]-[33]. A study in Xi’an, China including 372 HFMD cases found that exclusive breastfeeding was negatively associated with severity of HFMD [12]. One study from UK estimated that about half of hospitalizations for diarrhea and 27% of lower respiratory tract infections could be prevented if all infants were exclusively breastfed [27]. A large randomized clinical trial in Belarus found that infants who continue exclusive breastfeeding for 6 months or more appeared to have a significantly reduced risk of one or more episodes of gastrointestinal infection, with adjusted relative risk of 0.61 (95% CI: 0.41 to 0.93), which was similar with our finding [34]. Kramer and Kakuma’s systematic review suggested that infants who were exclusively breastfed for up to 6 months experienced less morbidity from gastrointestinal infection than those who were mixed breastfed [18].

This study suggested that the beneficial effect of exclusive breastfeeding could persist for 28 months, which was consistent with a few previous studies. For example, a comprehensive meta-analysis showed that breastfeeding substantially lowers the risk of death from infectious diseases in the first two years of life [16]. Lamberti et al. [35] evaluated the effect of breastfeeding on morbidity and mortality from diarrhea. Among infants younger than 6 months, the relative risk of dying from diarrhea was 10.5 (95% CI: 2.79-39.6) times higher among those infants who were not breastfed than those who were exclusively breastfed., among children aged between 6 and 23 months, the protective effect of breastfeeding was smaller, but still statistically significant (RR = 2.18, 95% CI: 1.44-4.16).

On the other hand, some studies have reported inconsistent findings, for instance, based on evidence from the United Kingdom Millennium Cohort, Quigley et al. [27] reported that the protective effect of breastfeeding against diarrhea and lower respiratory tract infection weared off soon after breastfeeding cessation. And a study from Spain found that exclusive breastfeeding for more than 4 months could protect children younger than 1 year from gastroenteritis infection [36]. However, that study followed up the children for only 1 year, limiting their ability to further explore the protective effect for longer time.

A systematic review by Kramer and coauthors concluded that exclusive breastfeeding during the first 6 months of life could decrease morbidity from gastrointestinal and allergic diseases [18],[37]. It has been therefore recommended by WHO that in the first 6 months of life, every child should be exclusively breastfed [38]. However, in discord with the WHO recommendation, just 45.3% had breastfed their child for at least 4 months, and only 21.6% had breastfed for 6 months in China [39]. There were a few barriers to consistent breastfeeding. For example, according to Zhang, et al’s study in 10 Chinese communities, some mothers (32.0%) considered powered milk as more nutritious than breast milk, and 27.0% of the mothers thought that breastfeeding was harmful to their body recovery [39], as a result, women were less inclined to breastfeed and were more likely to turn to formula where available.

Child care or kindergarten attendance is a known factor for hand, foot and mouth disease in the early years of childhood, as its environment could facilitate the transmission of infectious agents [14]. It was not a concern in this study, as we only included the children without attendance at kindergartens/child care centers. Household income and crowdness of the living environment were also possible confounding factors [15], however, in the current study, there was not significant difference between cases and controls, even when we included these factors in the model, the result estimates remained stable. We also tried to control for other potential confounding factors, such as hand-washing habits, playing with other children in playground, and going to the clinics, and the results in the multivariate analysis did not change substantially, suggesting the observed protective effect was independent of these confounding factors.

A few mechanisms for a possible protective effect of breastfeeding have been proposed. Maternal milk may confer several effects on the development of the gastroenteritis tract and its subsequent ability to fight against some infection and illness. Transfer of specific nutritional, immunoregulatory, immunomodulatory agents and anti-inflammatory components, particularly some antibodies produced by the mothers who have been exposed to such pathogens, may play an important role by promoting the maturation of infant immune competence [40],[41]. It was also possible that breast feeding itself may have the same effect on the production of proinflammatory agents through reducing discomfort and obtaining emotional support through the intimate contacts with their mothers [42]. Furthermore, some studies have suggested that breast feeding could increase nutrition intake which was adversely correlated with proinflammatory interleukin 1β and tumor necrosis factor α [43], and the breast feeding was also associated with the physical development of the children [12],[44]. This might partly help to explain the beneficial effect of breast feeding in this study. In addition, breastfed children might have fewer opportunities of exposure to the virus from contaminated milk formula, other liquids and complementary foods [45].

The main strength of our study was the large sample size that was representative of the target population. The findings from this study have some practical significance, providing the evidence of benefits of exclusive breastfeeding from Chinese population, and supporting the WHO’s recommendation that all infants should be exclusively breastfed at the first half year of their life. However, a recent survey found that the exclusive breastfeeding rate among children in the study area (36.2%) was lower than the national average [46], so more promotional and educational activities are needed to improve the knowledge and awareness of the public.

On the other hand, a few limitations should be considered. Selection bias might be a concern. Though we tried to select our subjects in a random selection manner, selection bias was possible, and our subjects were recruited from 6 cities of Guangdong Province, which might not represent the HFMD cases in this province, however our additional analysis found that the demographical characteristics of the HFMD cases in these 6 cities were similar with other areas of Guangdong Province, suggesting that the selection bias should be minimal. Some important information, such as breastfeeding months of the children, was not included in this survey and limited our study to provide further evidence of the association between breastfeeding and HFMD, particularly, lacking of this information did not allow us to examine whether the continuous protective effect of the exclusive breastfeeding for up to 28 months was due to persist breastfeeding after 6 months. Recall bias should not distort our results to a great extent. The present study used parental recall of breastfeeding type and other potential confounding factors of the subjects. The accuracy of the recall has been not assessed for the study population; however previous studies have suggested that the maternal reporting of breast feeding type was reliable up to several years after the birth of the infants [47]. And we tried to introduce this study to the cases and controls as a general health study. Furthermore most interviewers and participates did not know the linkage between breast feeding and this disease, so it was unlikely that the cases and controls had different recalling accuracy of their breastfeeding status. And most of the cases in this study were diagnosed based on clinical symptoms. The parents and guardians were usually reluctant to accept the virological test because of the discomfort to their children by blood collection. This may affect the reliability of the analysis. Therefore, future studies with both clinically and virologically diagnosed HFMD cases, particularly prospective cohort studies, are necessary to confirm the findings from this study.

## Conclusions

In conclusion, this study suggests that exclusive breastfeeding might have protective effect against hand, foot, and mouth disease among the children within 28 months of age, however, due to the potential limitations, more studies are warranted in the future studies.

## Authors’ original submitted files for images

Below are the links to the authors’ original submitted files for images. Authors’ original file for figure 1

## References

[1] World Health Organisation Staff (2003). Global Strategy for Infant and Young Child Feeding.

[2] Lucas A (1998). Programming by early nutrition: an experimental approach. J Nutr.

[3] Howie PW, Forsyth JS, Ogston SA, Clark A, Florey C (1990). Protective effect of breast feeding against infection. BMJ.

[4] Mao LX, Wu B, Bao WX, Han FA, Xu L, Ge QJ, Yang J, Yuan ZH, Miao CH, Huang XX, Zhang C, Xu H (2010). Epidemiology of hand, foot, and mouth disease and genotype characterization of Enterovirus 71 in Jiangsu, China. J Clin Virol.

[5] Chen KT, Chang HL, Wang ST, Cheng YT, Yang JY (2007). Epidemiologic features of hand-foot-mouth disease and herpangina caused by enterovirus 71 in Taiwan, 1998–2005. Pediatrics.

[6] Hubiche T, Schuffenecker I, Boralevi F, Léauté-Labrèze C, Bornebusch L, Chiaverini C, Phan A, Maruani A, Miquel J, Lafon M-E (2014). Dermatological spectrum of hand, foot, and mouth disease from classical to generalized exanthema. Pediatr Infect Dis J.

[7] Wu H, Wang H, Wang Q, Xin Q, Lin H (2014). The effect of meteorological factors on adolescent hand, foot, and mouth disease and associated effect modifiers. Global Health Action.

[8] Jin Y, Zhang J, Sun J-L, Chang Z-R (2012). Epidemiology of hand, foot and mouth disease in mainland of China, 2011. Disease Surveillance.

[9] Ho M, Chen ER, Hsu KH, Twu SJ, Chen KT, Tsai SF, Wang JR, Shih SR (1999). An epidemic of enterovirus 71 infection in Taiwan. N Engl J Med.

[10] Hii YL, Rocklov J, Ng N (2011). Short term effects of weather on hand, foot and mouth disease. PLoS One.

[11] Ma E, Wong S, Wong C, Chuang SK, Tsang T (2011). Effects of public health interventions in reducing transmission of hand, foot, and mouth disease. Pediatr Infect Dis J.

[12] Zhu Q, Li Y, Li N, Han Q, Liu Z, Li Z, Qiu J, Zhang G, Li F, Tian N (2012). Prolonged exclusive breastfeeding, autumn birth and increased gestational age are associated with lower risk of fever in children with hand, foot, and mouth disease. Eur J Clin Microbiol Infect Dis.

[13] Lin HL, Zou H, Nie J, Liu CX, Li ZJ (2013). Short term effects of El Nino-Southern Oscillation on hand, foot, and mouth disease in Shenzhen, China. PLoS One.

[14] Chang LY, King CC, Hsu KH, Ning HC, Tsao KC, Li CC, Huang YC, Shih SR, Chiou ST, Chen PY (2002). Risk factors of enterovirus 71 infection and associated hand, foot, and mouth disease/herpangina in children during an epidemic in Taiwan. Pediatrics.

[15] Ruan F, Yang T, Ma H, Jin Y, Song S, Fontaine RE, Zhu B-P (2011). Risk factors for hand, foot, and mouth disease and herpangina and the preventive effect of hand-washing. Pediatrics.

[16] WHO Collaborative Study Team on the Role of Breastfeeding on the Prevention of Infant Mortality (2000). Effect of breastfeeding on infant and child mortality due to infectious diseases in less developed countries: a pooled analysis. Lancet.

[17] Bahl R, Frost C, Kirkwood BR, Edmond K, Martines J, Bhandari N, Arthur P (2005). Infant feeding patterns and risks of death and hospitalization in the first half of infancy: multicentre cohort study. Bull World Health Organ.

[18] Kakuma R (2002). The Optimal Duration of Exclusive Breastfeeding: A Systematic Review.

[19] Kramer MS, Guo T, Platt RW, Sevkovskaya Z, Dzikovich I, Collet J-P, Shapiro S, Chalmers B, Hodnett E, Vanilovich I (2003). Infant growth and health outcomes associated with 3 compared with 6 mo of exclusive breastfeeding. The American Journal of Clinical Nutrition.

[20] Ministry of Health of the People’s Republic of China: *Hand, Foot and Mouth Disease Clinic Guide (2010 edition).* Beijing: 2010.

[21] World Health Organization (2011). A Guide to Clinical Management and Public Health Response for Hand, Foot and Mouth Disease (HFMD).

[22] Li W, Yi L, Su J, Lu J, Zeng H, Guan D, Ma C, Zhang W, Xiao H, Li H, Zhang Y, Lin J, Ke C (2013). Seroepidemiology of human enterovirus71 and coxsackievirusA16 among children in Guangdong province, China. BMC Infect Dis.

[23] Ang LW, Koh BK, Chan KP, Chua LT, James L, Goh KT (2009). Epidemiology and control of hand, foot and mouth disease in Singapore. Ann Acad Med Singapore.

[24] Chen C, Lin H, Li X, Lang L, Xiao X, Ding P, He P, Zhang Y, Wang M, Liu Q (2014). Short-term effects of meteorological factors on children hand, foot and mouth disease in Guangzhou, China. Int J Biometeorol.

[25] Lin H, Ng S, Chan S, Chan WM, Lee KC, Ho SC, Tian L (2011). Institutional risk factors for norovirus outbreaks in Hong Kong elderly homes: a retrospective cohort study. BMC Public Health.

[26] Ihaka R, Gentleman R (1996). R: A language for data analysis and graphics. J Comput Graph Stat.

[27] Quigley MA, Kelly YJ, Sacker A (2007). Breastfeeding and hospitalization for diarrheal and respiratory infection in the United Kingdom Millennium Cohort Study. Pediatrics.

[28] Pisacane A, Graziano L, Zona G, Granata G, Dolezalova H, Cafiero M, Coppola A, Scarpellino B, Ummarino M, Mazzarella G (1994). Breast feeding and acute lower respiratory infection. Acta Paediatr.

[29] Oddy W, Sly P, De Klerk N, Landau L, Kendall G, Holt P, Stanley F (2003). Breast feeding and respiratory morbidity in infancy: a birth cohort study. Arch Dis Child.

[30] Levine OS, Farley M, Harrison LH, Lefkowitz L, McGeer A, Schwartz B (1999). Risk factors for invasive pneumococcal disease in children: a population-based case–control study in North America. Pediatrics.

[31] Chen Y, Yu S, Li W-x (1988). Artificial feeding and hospitalization in the first 18 months of life. Pediatrics.

[32] Chantry CJ, Howard CR, Auinger P (2006). Full breastfeeding duration and associated decrease in respiratory tract infection in US children. Pediatrics.

[33] Rebhan B, Kohlhuber M, Schwegler U, Fromme H, Abou‐Dakn M, Koletzko BV (2009). Breastfeeding duration and exclusivity associated with infants’ health and growth: data from a prospective cohort study in Bavaria, Germany. Acta Paediatr.

[34] Koletzko B, Michaelsen KF, Hernell O: *Short and Long Term Effects of Breast Feeding on Child Health, Advances in Experimental Medicine and Biology Vol. 478.*Springer; 2000.

[35] Lamberti LM, Walker CLF, Noiman A, Victora C, Black RE (2011). Breastfeeding and the risk for diarrhea morbidity and mortality. BMC Public Health.

[36] Talayero JMP, Lizán-García M, Puime ÁO, Muncharaz MJB, Soto BB, Sánchez-Palomares M, Serrano LS, Rivera LL (2006). Full breastfeeding and hospitalization as a result of infections in the first year of life. Pediatrics.

[37] Kramer MS, Kakuma R (2004). The optimal duration of exclusive breastfeeding: a systematic review. Adv Exp Med Biol.

[38] World Health Organization: Learning from large-scale community-based programmes to improve breastfeeding practices. http://www.who.int/nutrition/publications/infantfeeding/9789241597371/en/index.html. Accessed 20 July 2011.,

[39] Zhang W, Hao B, Wang L (2004). Situation of breastfeeding in ten cities in five provinces in China. Chinese Journal of Health Education.

[40] Jackson KM, Nazar AM (2006). Breastfeeding, the immune response, and long-term health. JAOA: Journal of the American Osteopathic Association.

[41] Hanson L (1999). Human milk and host defence: immediate and long‐term effects. Acta Paediatr.

[42] Pisacane A, Continisio P, Palma O, Cataldo S, De Michele F, Vairo U (2010). Breastfeeding and risk for fever after immunization. Pediatrics.

[43] López-Alarcón M, Garza C, Habicht J-P, Martínez L, Pegueros V, Villalpando S (2002). Breastfeeding attenuates reductions in energy intake induced by a mild immunologic stimulus represented by DPTH immunization: possible roles of interleukin-1β, tumor necrosis factor-α and leptin. The Journal of nutrition.

[44] Anderson AK (2009). Association between infant feeding and early postpartum infant body composition: a pilot prospective study. International journal of pediatrics.

[45] Long KZ, Wood JW, Gariby EV, Weiss KM, Mathewson JJ, Francisco J, DuPont HL, Wilson RA (1994). Proportional hazards analysis of diarrhea due to enterotoxigenic Escherichia coli and breast feeding in a cohort of urban Mexican children. Am J Epidemiol.

[46] Chun SY (2006). Investigation of Growth and Feeding Status of 0–3 Years Old Young Children of Tianhe Region in Guangzhou.

[47] Li R, Scanlon KS, Serdula MK (2005). The validity and reliability of maternal recall of breastfeeding practice. Nutr Rev.

