# Supplementary figures and images for: Protective effect of exclusive breastfeeding against hand, foot and mouth disease
Source: BMC Infect Dis. 2014 Dec 4;14:645. doi: 10.1186/s12879-014-0645-6 (PMC4273484; doi:10.1186/s12879-014-0645-6)

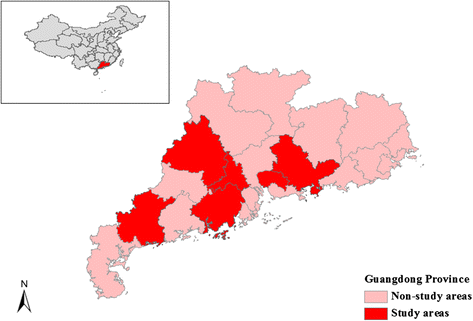

Supplement: Supplementary file 1 — Authors’ original file for figure 1 [file 12879_2014_645_MOESM1_ESM.gif]
